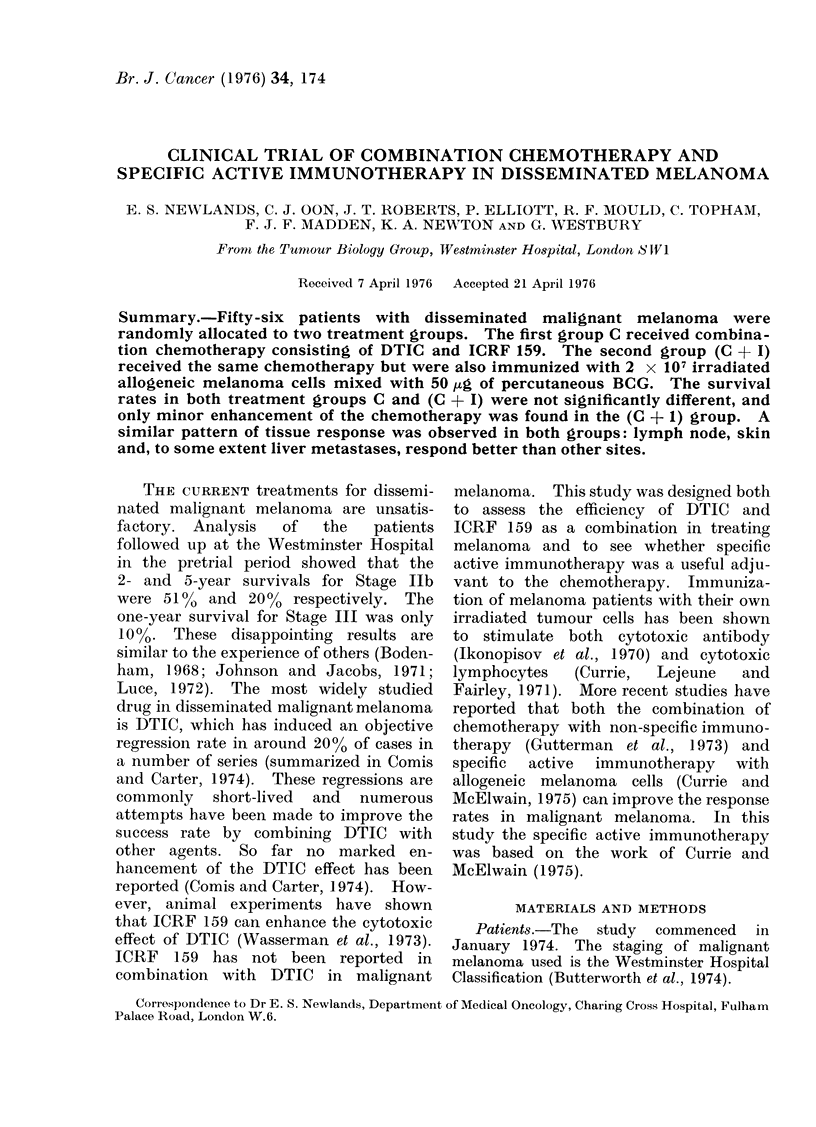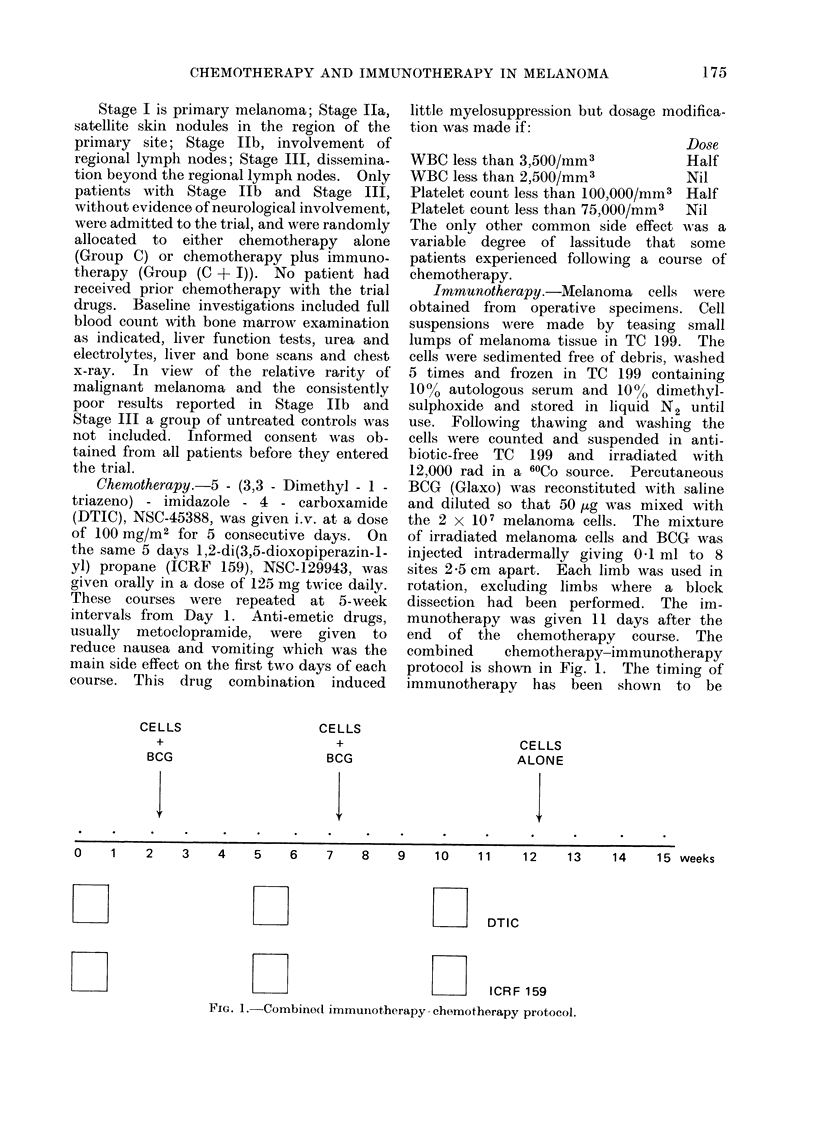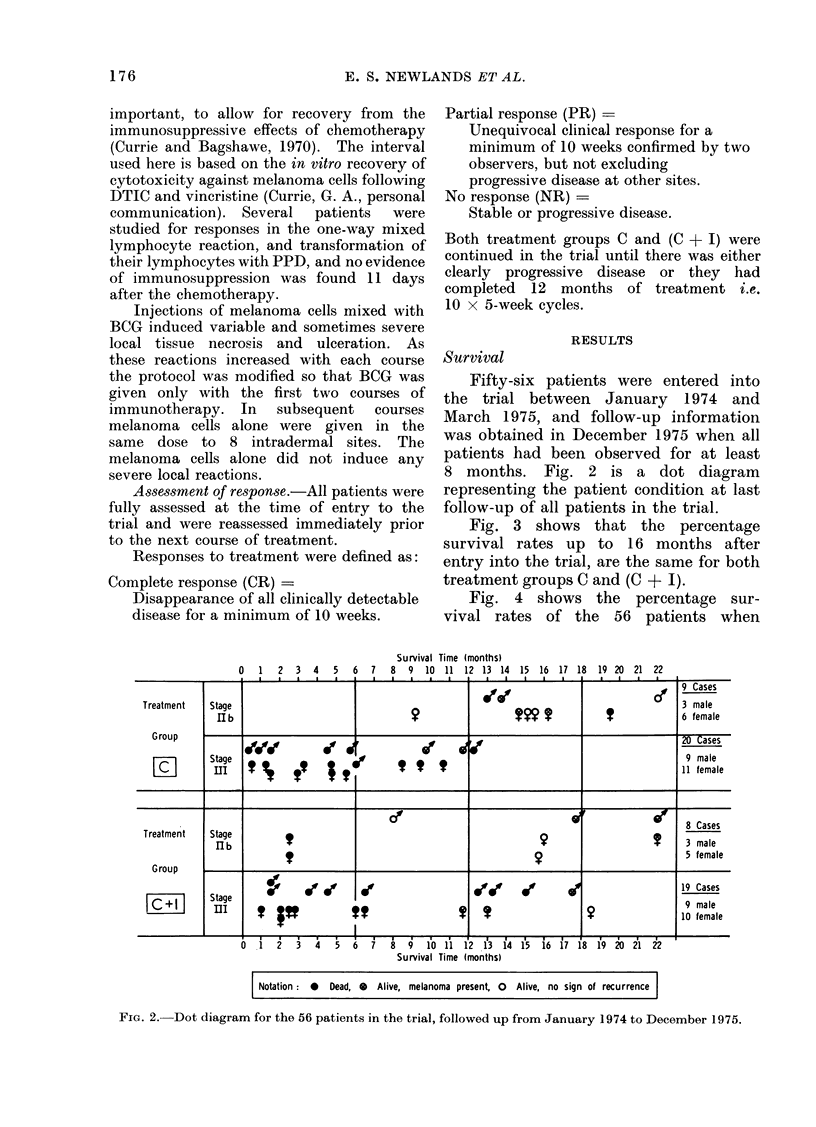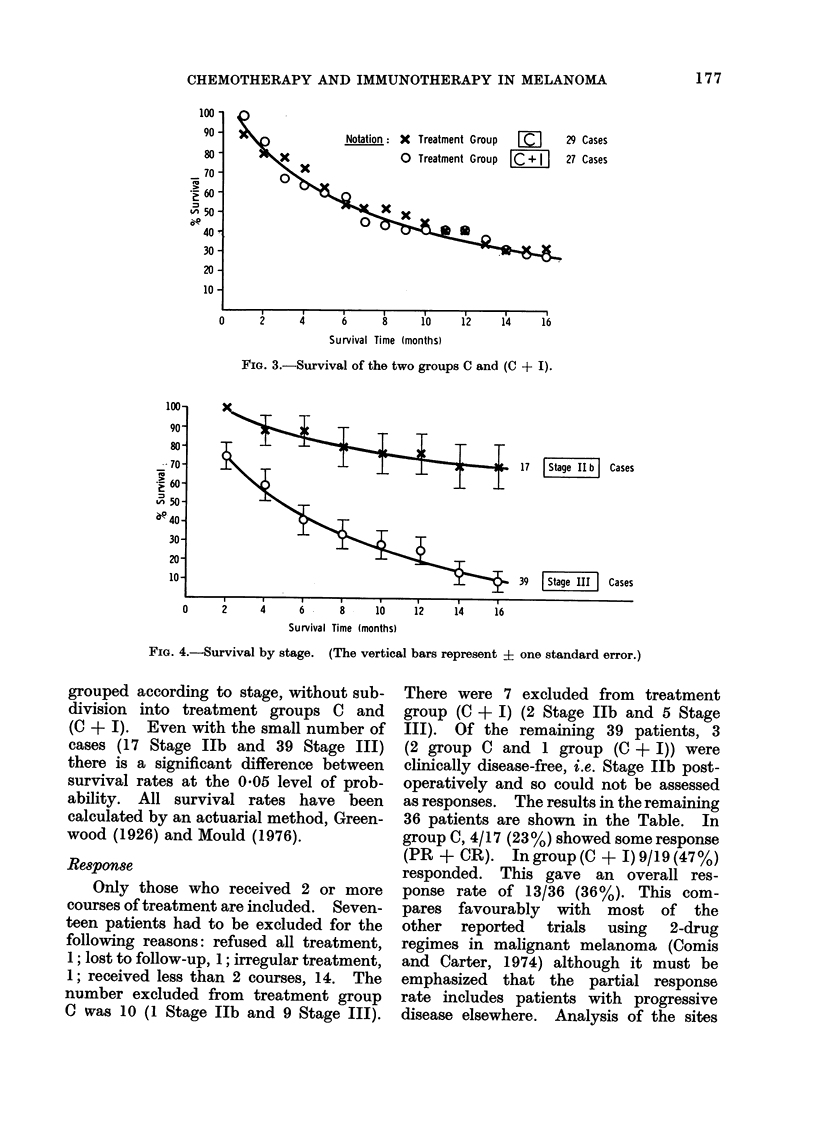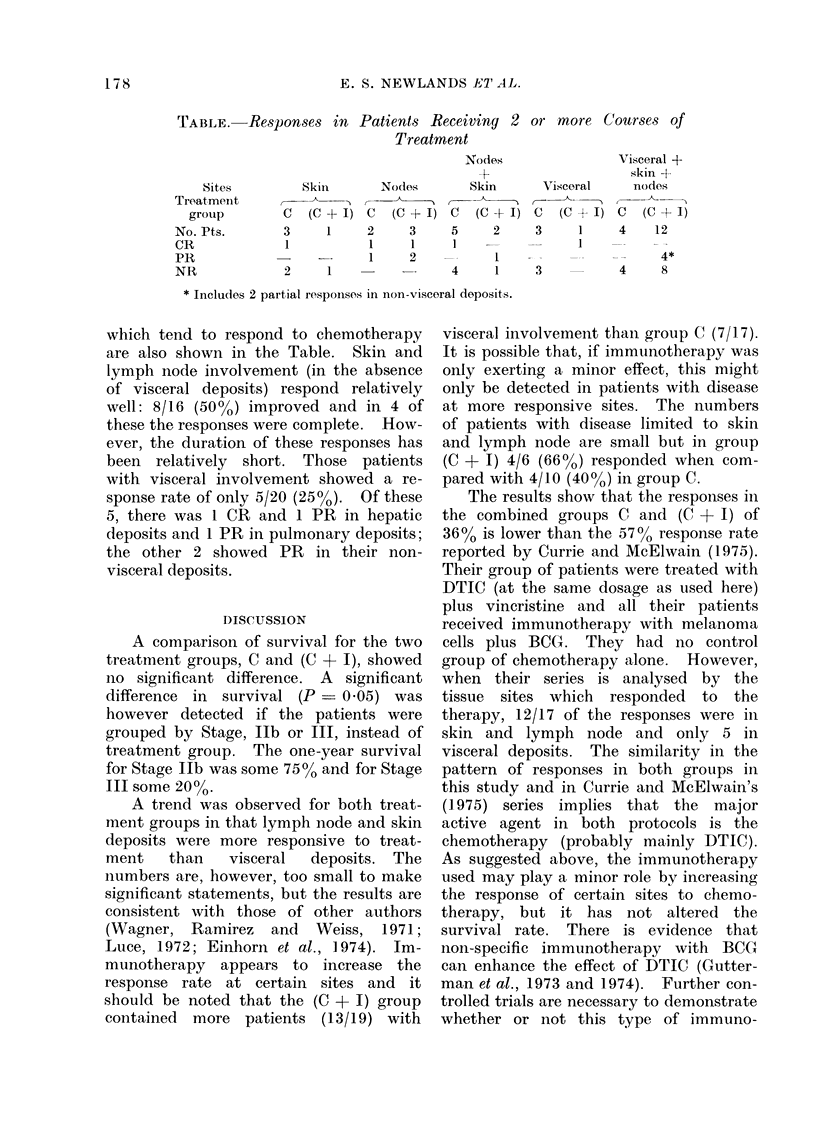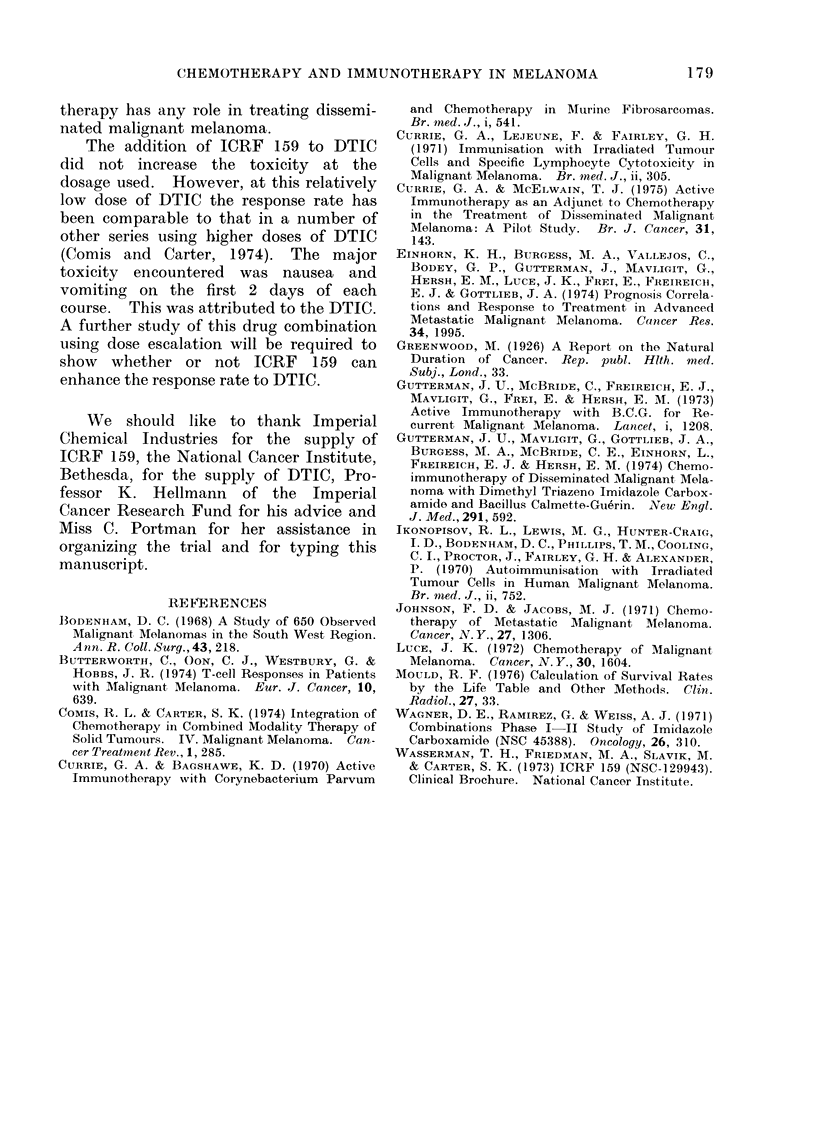# Clinical trial of combination chemotherapy and specific active immunotherapy in disseminated melanoma.

**DOI:** 10.1038/bjc.1976.140

**Published:** 1976-08

**Authors:** E. S. Newlands, C. J. Oon, J. T. Roberts, P. Elliott, R. F. Mould, C. Topham, F. J. Madden, K. A. Newton, G. Westbury

## Abstract

Fifty-six patients with disseminated malignant melanoma were randomly allocated to two treatment groups. The first group C received combination chemotherapy consisting of DTIC and ICRF 159. The second group (C+I) received the same chemotherapy but were also immunized with 2 X 10(7) irradiated allogeneic melanoma cells mixed with 50 mug of percutaneous BCG. The survival rates in both treatment groups C and (C+I) were not significantly different, and only minor enhancement of the chemotherapy was found in the (C+I) group. A similar pattern of tissue response was observed in both groups: lymph node, skin and, to some extent liver metastases, respond better than other sites.


*Br. J. Cancer* (1976) **34**, 174

### CLINICAL TRIAL OF COMBINATION CHEMOTHERAPY AND SPECIFIC ACTIVE IMMUNOTHERAPY IN DISSEMINATED MELANOMA

E. S. NEWLANDS, C. J. OON, J. T. ROBERTS, P. ELLIOTT, R. F. MOULD, C. TOPHAM, F. J. F. MADDEN, K. A. NEWTON AND G. WESTBURY

*From the Tumour Biology Group, Westminster Hospital, London SW1*

Received 7 April 1976 Accepted 21 April 1976

**Summary.—Fifty-six patients with disseminated malignant melanoma were randomly allocated to two treatment groups. The first group C received combination chemotherapy consisting of DTIC and ICRF 159. The second group (C + I) received the same chemotherapy but were also immunized with 2 $\times$ $10^7$ irradiated allogeneic melanoma cells mixed with 50 $\mu$g of percutaneous BCG. The survival rates in both treatment groups C and (C + I) were not significantly different, and only minor enhancement of the chemotherapy was found in the (C + 1) group. A similar pattern of tissue response was observed in both groups: lymph node, skin and, to some extent liver metastases, respond better than other sites.**

THE CURRENT treatments for disseminated malignant melanoma are unsatisfactory. Analysis of the patients followed up at the Westminster Hospital in the pretrial period showed that the 2- and 5-year survivals for Stage IIb were 51% and 20% respectively. The one-year survival for Stage III was only 10%. These disappointing results are similar to the experience of others (Bodenham, 1968; Johnson and Jacobs, 1971; Luce, 1972). The most widely studied drug in disseminated malignant melanoma is DTIC, which has induced an objective regression rate in around 20% of cases in a number of series (summarized in Comis and Carter, 1974). These regressions are commonly short-lived and numerous attempts have been made to improve the success rate by combining DTIC with other agents. So far no marked enhancement of the DTIC effect has been reported (Comis and Carter, 1974). However, animal experiments have shown that ICRF 159 can enhance the cytotoxic effect of DTIC (Wasserman *et al.*, 1973). ICRF 159 has not been reported in combination with DTIC in malignant melanoma. This study was designed both to assess the efficiency of DTIC and ICRF 159 as a combination in treating melanoma and to see whether specific active immunotherapy was a useful adjuvant to the chemotherapy. Immunization of melanoma patients with their own irradiated tumour cells has been shown to stimulate both cytotoxic antibody (Ikonopisov *et al.*, 1970) and cytotoxic lymphocytes (Currie, Lejeune and Fairley, 1971). More recent studies have reported that both the combination of chemotherapy with non-specific immunotherapy (Gutterman *et al.*, 1973) and specific active immunotherapy with allogeneic melanoma cells (Currie and McElwain, 1975) can improve the response rates in malignant melanoma. In this study the specific active immunotherapy was based on the work of Currie and McElwain (1975).

### MATERIALS AND METHODS

*Patients*.—The study commenced in January 1974. The staging of malignant melanoma used is the Westminster Hospital Classification (Butterworth *et al.*, 1974).

Correspondence to Dr E. S. Newlands, Department of Medical Oncology, Charing Cross Hospital, Fulham Palace Road, London W.6.

Stage I is primary melanoma; Stage IIa, satellite skin nodules in the region of the primary site; Stage IIb, involvement of regional lymph nodes; Stage III, dissemination beyond the regional lymph nodes. Only patients with Stage IIb and Stage III, without evidence of neurological involvement, were admitted to the trial, and were randomly allocated to either chemotherapy alone (Group C) or chemotherapy plus immunotherapy (Group (C + I)). No patient had received prior chemotherapy with the trial drugs. Baseline investigations included full blood count with bone marrow examination as indicated, liver function tests, urea and electrolytes, liver and bone scans and chest x-ray. In view of the relative rarity of malignant melanoma and the consistently poor results reported in Stage IIb and Stage III a group of untreated controls was not included. Informed consent was obtained from all patients before they entered the trial.

*Chemotherapy*.—5 - (3,3 - Dimethyl - 1 - triazeno) - imidazole - 4 - carboxamide (DTIC), NSC-45388, was given i.v. at a dose of 100 mg/m$^2$ for 5 consecutive days. On the same 5 days 1,2-di(3,5-dioxopiperazin-1-yl) propane (ICRF 159), NSC-129943, was given orally in a dose of 125 mg twice daily. These courses were repeated at 5-week intervals from Day 1. Anti-emetic drugs, usually metoclopramide, were given to reduce nausea and vomiting which was the main side effect on the first two days of each course. This drug combination induced little myelosuppression but dosage modification was made if:

| | *Dose* |
|---|---|
| WBC less than 3,500/mm$^3$ | Half |
| WBC less than 2,500/mm$^3$ | Nil |
| Platelet count less than 100,000/mm$^3$ | Half |
| Platelet count less than 75,000/mm$^3$ | Nil |

The only other common side effect was a variable degree of lassitude that some patients experienced following a course of chemotherapy.

*Immunotherapy*.—Melanoma cells were obtained from operative specimens. Cell suspensions were made by teasing small lumps of melanoma tissue in TC 199. The cells were sedimented free of debris, washed 5 times and frozen in TC 199 containing 10% autologous serum and 10% dimethylsulphoxide and stored in liquid $N_2$ until use. Following thawing and washing the cells were counted and suspended in antibiotic-free TC 199 and irradiated with 12,000 rad in a $^{60}$Co source. Percutaneous BCG (Glaxo) was reconstituted with saline and diluted so that 50 $\mu$g was mixed with the 2 $\times$ 10$^7$ melanoma cells. The mixture of irradiated melanoma cells and BCG was injected intradermally giving 0·1 ml to 8 sites 2·5 cm apart. Each limb was used in rotation, excluding limbs where a block dissection had been performed. The immunotherapy was given 11 days after the end of the chemotherapy course. The combined chemotherapy–immunotherapy protocol is shown in Fig. 1. The timing of immunotherapy has been shown to be

FIG. 1.—Combined immunotherapy-chemotherapy protocol.

important, to allow for recovery from the immunosuppressive effects of chemotherapy (Currie and Bagshawe, 1970). The interval used here is based on the *in vitro* recovery of cytotoxicity against melanoma cells following DTIC and vincristine (Currie, G. A., personal communication). Several patients were studied for responses in the one-way mixed lymphocyte reaction, and transformation of their lymphocytes with PPD, and no evidence of immunosuppression was found 11 days after the chemotherapy.

Injections of melanoma cells mixed with BCG induced variable and sometimes severe local tissue necrosis and ulceration. As these reactions increased with each course the protocol was modified so that BCG was given only with the first two courses of immunotherapy. In subsequent courses melanoma cells alone were given in the same dose to 8 intradermal sites. The melanoma cells alone did not induce any severe local reactions.

*Assessment of response.*—All patients were fully assessed at the time of entry to the trial and were reassessed immediately prior to the next course of treatment.

Responses to treatment were defined as:

Complete response (CR) =
Disappearance of all clinically detectable disease for a minimum of 10 weeks.

Partial response (PR) =
Unequivocal clinical response for a minimum of 10 weeks confirmed by two observers, but not excluding progressive disease at other sites.

No response (NR) =
Stable or progressive disease.

Both treatment groups C and (C + I) were continued in the trial until there was either clearly progressive disease or they had completed 12 months of treatment *i.e.* 10 × 5-week cycles.

### RESULTS

### *Survival*

Fifty-six patients were entered into the trial between January 1974 and March 1975, and follow-up information was obtained in December 1975 when all patients had been observed for at least 8 months. Fig. 2 is a dot diagram representing the patient condition at last follow-up of all patients in the trial.

Fig. 3 shows that the percentage survival rates up to 16 months after entry into the trial, are the same for both treatment groups C and (C + I).

Fig. 4 shows the percentage survival rates of the 56 patients when

FIG. 2.—Dot diagram for the 56 patients in the trial, followed up from January 1974 to December 1975.

FIG. 3.—Survival of the two groups C and (C + I).

FIG. 4.—Survival by stage. (The vertical bars represent ± one standard error.)

grouped according to stage, without subdivision into treatment groups C and (C + I). Even with the small number of cases (17 Stage IIb and 39 Stage III) there is a significant difference between survival rates at the 0·05 level of probability. All survival rates have been calculated by an actuarial method, Greenwood (1926) and Mould (1976).

*Response*

Only those who received 2 or more courses of treatment are included. Seventeen patients had to be excluded for the following reasons: refused all treatment, 1; lost to follow-up, 1; irregular treatment, 1; received less than 2 courses, 14. The number excluded from treatment group C was 10 (1 Stage IIb and 9 Stage III). There were 7 excluded from treatment group (C + I) (2 Stage IIb and 5 Stage III). Of the remaining 39 patients, 3 (2 group C and 1 group (C + I)) were clinically disease-free, *i.e.* Stage IIb postoperatively and so could not be assessed as responses. The results in the remaining 36 patients are shown in the Table. In group C, 4/17 (23%) showed some response (PR + CR). In group (C + I) 9/19 (47%) responded. This gave an overall response rate of 13/36 (36%). This compares favourably with most of the other reported trials using 2-drug regimes in malignant melanoma (Comis and Carter, 1974) although it must be emphasized that the partial response rate includes patients with progressive disease elsewhere. Analysis of the sites

TABLE.—*Responses in Patients Receiving 2 or more Courses of Treatment*

| Sites | Skin | | Nodes | | Nodes + Skin | | Visceral | | Visceral + skin + nodes | |
|---|---|---|---|---|---|---|---|---|---|---|
| Treatment group | C | (C + I) | C | (C + I) | C | (C + I) | C | (C + I) | C | (C + I) |
| No. Pts. | 3 | 1 | 2 | 3 | 5 | 2 | 3 | 1 | 4 | 12 |
| CR | 1 | | 1 | 1 | 1 | — | — | 1 | — | — |
| PR | — | — | 1 | 2 | — | 1 | — | — | — | 4* |
| NR | 2 | 1 | — | — | 4 | 1 | 3 | — | 4 | 8 |

* Includes 2 partial responses in non-visceral deposits.

which tend to respond to chemotherapy are also shown in the Table. Skin and lymph node involvement (in the absence of visceral deposits) respond relatively well: 8/16 (50%) improved and in 4 of these the responses were complete. However, the duration of these responses has been relatively short. Those patients with visceral involvement showed a response rate of only 5/20 (25%). Of these 5, there was 1 CR and 1 PR in hepatic deposits and 1 PR in pulmonary deposits; the other 2 showed PR in their non-visceral deposits.

### DISCUSSION

A comparison of survival for the two treatment groups, C and (C + I), showed no significant difference. A significant difference in survival ($P = 0{\cdot}05$) was however detected if the patients were grouped by Stage, IIb or III, instead of treatment group. The one-year survival for Stage IIb was some 75% and for Stage III some 20%.

A trend was observed for both treatment groups in that lymph node and skin deposits were more responsive to treatment than visceral deposits. The numbers are, however, too small to make significant statements, but the results are consistent with those of other authors (Wagner, Ramirez and Weiss, 1971; Luce, 1972; Einhorn *et al.*, 1974). Immunotherapy appears to increase the response rate at certain sites and it should be noted that the (C + I) group contained more patients (13/19) with visceral involvement than group C (7/17). It is possible that, if immunotherapy was only exerting a minor effect, this might only be detected in patients with disease at more responsive sites. The numbers of patients with disease limited to skin and lymph node are small but in group (C + I) 4/6 (66%) responded when compared with 4/10 (40%) in group C.

The results show that the responses in the combined groups C and (C + I) of 36% is lower than the 57% response rate reported by Currie and McElwain (1975). Their group of patients were treated with DTIC (at the same dosage as used here) plus vincristine and all their patients received immunotherapy with melanoma cells plus BCG. They had no control group of chemotherapy alone. However, when their series is analysed by the tissue sites which responded to the therapy, 12/17 of the responses were in skin and lymph node and only 5 in visceral deposits. The similarity in the pattern of responses in both groups in this study and in Currie and McElwain's (1975) series implies that the major active agent in both protocols is the chemotherapy (probably mainly DTIC). As suggested above, the immunotherapy used may play a minor role by increasing the response of certain sites to chemotherapy, but it has not altered the survival rate. There is evidence that non-specific immunotherapy with BCG can enhance the effect of DTIC (Gutterman *et al.*, 1973 and 1974). Further controlled trials are necessary to demonstrate whether or not this type of immunotherapy has any role in treating disseminated malignant melanoma.

The addition of ICRF 159 to DTIC did not increase the toxicity at the dosage used. However, at this relatively low dose of DTIC the response rate has been comparable to that in a number of other series using higher doses of DTIC (Comis and Carter, 1974). The major toxicity encountered was nausea and vomiting on the first 2 days of each course. This was attributed to the DTIC. A further study of this drug combination using dose escalation will be required to show whether or not ICRF 159 can enhance the response rate to DTIC.

We should like to thank Imperial Chemical Industries for the supply of ICRF 159, the National Cancer Institute, Bethesda, for the supply of DTIC, Professor K. Hellmann of the Imperial Cancer Research Fund for his advice and Miss C. Portman for her assistance in organizing the trial and for typing this manuscript.

### REFERENCES

BODENHAM, D. C. (1968) A Study of 650 Observed Malignant Melanomas in the South West Region. *Ann. R. Coll. Surg.*, **43**, 218.

BUTTERWORTH, C., OON, C. J., WESTBURY, G. & HOBBS, J. R. (1974) T-cell Responses in Patients with Malignant Melanoma. *Eur. J. Cancer*, **10**, 639.

COMIS, R. L. & CARTER, S. K. (1974) Integration of Chemotherapy in Combined Modality Therapy of Solid Tumours. IV. Malignant Melanoma. *Cancer Treatment Rev.*, **1**, 285.

CURRIE, G. A. & BAGSHAWE, K. D. (1970) Active Immunotherapy with Corynebacterium Parvum and Chemotherapy in Murine Fibrosarcomas. *Br. med. J.*, i, 541.

CURRIE, G. A., LEJEUNE, F. & FAIRLEY, G. H. (1971) Immunisation with Irradiated Tumour Cells and Specific Lymphocyte Cytotoxicity in Malignant Melanoma. *Br. med. J.*, ii, 305.

CURRIE, G. A. & MCELWAIN, T. J. (1975) Active Immunotherapy as an Adjunct to Chemotherapy in the Treatment of Disseminated Malignant Melanoma: A Pilot Study. *Br. J. Cancer*, **31**, 143.

EINHORN, K. H., BURGESS, M. A., VALLEJOS, C., BODEY, G. P., GUTTERMAN, J., MAVLIGIT, G., HERSH, E. M., LUCE, J. K., FREI, E., FREIREICH, E. J. & GOTTLIEB, J. A. (1974) Prognosis Correlations and Response to Treatment in Advanced Metastatic Malignant Melanoma. *Cancer Res.* **34**, 1995.

GREENWOOD, M. (1926) A Report on the Natural Duration of Cancer. *Rep. publ. Hlth. med. Subj., Lond.*, 33.

GUTTERMAN, J. U., MCBRIDE, C., FREIREICH, E. J., MAVLIGIT, G., FREI, E. & HERSH, E. M. (1973) Active Immunotherapy with B.C.G. for Recurrent Malignant Melanoma. *Lancet*, i, 1208.

GUTTERMAN, J. U., MAVLIGIT, G., GOTTLIEB, J. A., BURGESS, M. A., MCBRIDE, C. E., EINHORN, L., FREIREICH, E. J. & HERSH, E. M. (1974) Chemoimmunotherapy of Disseminated Malignant Melanoma with Dimethyl Triazeno Imidazole Carboxamide and Bacillus Calmette-Guérin. *New Engl. J. Med.*, **291**, 592.

IKONOPISOV, R. L., LEWIS, M. G., HUNTER-CRAIG, I. D., BODENHAM, D. C., PHILLIPS, T. M., COOLING, C. I., PROCTOR, J., FAIRLEY, G. H. & ALEXANDER, P. (1970) Autoimmunisation with Irradiated Tumour Cells in Human Malignant Melanoma. *Br. med. J.*, ii, 752.

JOHNSON, F. D. & JACOBS, M. J. (1971) Chemotherapy of Metastatic Malignant Melanoma. *Cancer, N.Y.*, **27**, 1306.

LUCE, J. K. (1972) Chemotherapy of Malignant Melanoma. *Cancer, N.Y.*, **30**, 1604.

MOULD, R. F. (1976) Calculation of Survival Rates by the Life Table and Other Methods. *Clin. Radiol.*, **27**, 33.

WAGNER, D. E., RAMIREZ, G. & WEISS, A. J. (1971) Combinations Phase I—II Study of Imidazole Carboxamide (NSC 45388). *Oncology*, **26**, 310.

WASSERMAN, T. H., FRIEDMAN, M. A., SLAVIK, M. & CARTER, S. K. (1973) ICRF 159 (NSC-129943). Clinical Brochure. National Cancer Institute.